# Meningioma: current updates on genetics, classification, and mouse modeling

**DOI:** 10.48101/ujms.v129.10579

**Published:** 2024-03-18

**Authors:** Frank Szulzewsky, H. Nayanga Thirimanne, Eric C. Holland

**Affiliations:** a Human Biology Division, Fred Hutchinson Cancer Center, Seattle, WA, USA; b Seattle Translational Tumor Research Center, Fred Hutchinson Cancer Center, Seattle, WA, USA

**Keywords:** Meningioma, brain tumor, YAP1, Hippo, TRAF7, KLF4, AKT, mouse modeling, RCAS, molecular classifications

## Abstract

Meningiomas, the most common primary brain tumors in adults, are often benign and curable by surgical resection. However, a subset is of higher grade, shows aggressive growth behavior as well as brain invasion, and often recurs even after several rounds of surgery. Increasing evidence suggests that tumor classification and grading primarily based on histopathology do not always accurately predict tumor aggressiveness and recurrence behavior. The underlying biology of aggressive treatment-resistant meningiomas and the impact of specific genetic aberrations present in these high-grade tumors is still only insufficiently understood. Therefore, an in-depth research into the biology of this tumor type is warranted. More recent studies based on large-scale molecular data such as whole exome/genome sequencing, DNA methylation sequencing, and RNA sequencing have provided new insights into the biology of meningiomas and have revealed new risk factors and prognostic subtypes. The most common genetic aberration in meningiomas is functional loss of NF2 and occurs in both low- and high-grade meningiomas, whereas NF2-wildtype meningiomas are enriched for recurrent mutations in TRAF7, KLF4, AKT1, PI3KCA, and SMO and are more frequently benign. Most meningioma mouse models are based on patient-derived xenografts and only recently have new genetically engineered mouse models of meningioma been developed that will aid in the systematic evaluation of specific mutations found in meningioma and their impact on tumor behavior. In this article, we review recent advances in the understanding of meningioma biology and classification and highlight the most common genetic mutations, as well as discuss new genetically engineered mouse models of meningioma.

## Overview

Although meningiomas are the most common primary brain tumors in adults, with an incidence of 9.51 per 100,000 (1), these tumors are an understudied disease, and we have only recently gained more in-depth insights into the genetic aberrations that drive these cancers. This might be attributable to the fact that the majority of meningiomas are benign, do not present with brain invasion, and are frequently curable by surgery alone. The scarcity of reliable experimental systems and genetically engineered mouse models (GEMMs) has furthermore hampered the development of novel targeted therapies. However, although only a minority of meningiomas are of higher-grade, atypical and anaplastic meningiomas frequently invade into the brain and tend to recur even after multiple rounds of surgery, chemo-, and radiation therapy, highlighting the need for a better understanding of the oncogenic (genetic or epigenetic) drivers of these tumors.

Meningiomas occur in all age groups but are significantly enriched in women and older adults >65 years of age (1). These tumors occur in a variety of anatomical locations (2, 3), with the most common being convexity meningiomas (growing on the surface of the brain directly under the skull), parasagittal and falcine meningiomas (forming in or next to the falx), and skull-base meningiomas (located near the bottom of the skull and in the back of the eyes). Less common are intraventricular meningiomas (originating from the lateral ventricles), tumors arising along the tentorium, and spinal meningiomas. Each of the locations is associated with specific mutational patterns and presents with a specific set of challenges that affect tumor behavior, treatment strategy, and ultimately prognosis. Skull-base meningiomas are generally more benign but can be less accessible to surgery due to their deep location and their frequent involvement of major blood vessels and cranial nerves, often preventing complete resection. By contrast, convexity/parasagittal meningiomas are generally easier to remove surgically but frequently tend to be more biologically aggressive and of higher-grade.

Meningiomas are thought to arise from meningothelial arachnoid cells of the meninges. In general, they are firmly attached to the inner surface of the dura and are well-circumscribed; however, higher-grade tumors can frequently show brain invasion. These tumors can show a wide variety of histopathological appearances, with 15 distinct variants recognized by the 2021 WHO classification (4). Historically, these tumors have been classified into three WHO grades, based on established histologic criteria, such as mitotic activity, brain invasion, hypercellularity, necrosis, and pattern-less growth. The majority (around 70%) of meningiomas are of WHO grade 1, while WHO grade 2 (around 28%) and grade 3 (around 3%) meningiomas are considerably rarer. In general, WHO grades 2 and 3 meningiomas have a higher likelihood of recurrence; however, the histological grade frequently does not accurately predict tumor growth, behavior, and recurrence, highlighting the need for additional classifications based on genetic, epigenetic, and transcriptional markers.

## Frequent genomic aberrations in meningioma

The most common genetic aberration in meningiomas is functional loss of NF2, the gene encoding Merlin, found in around 40 to 60% of meningiomas, in most cases due to loss of chromosome 22, inactivating point mutations, or gene fusions (5, 6). *NF2* wild-type meningiomas frequently harbor mutations in *TRAF7*, *KLF4*, *AKT1*, *PIK3CA*, and *SMO* and are enriched in skull-base meningiomas (7). Mutations in KLF4, AKT1, and PIK3CA frequently cooccur with TRAF7 mutations but are generally mutually exclusive from each other, suggesting that they may all achieve a similar outcome, namely, the activation of the PI3K-AKT-mTOR pathway. Higher-grade meningiomas occur in all molecular subgroups but are enriched in NF2 mutant meningiomas. While low-grade NF2 mutant meningiomas generally only exhibit loss of chromosome 22 (harboring the NF2 gene) and do not harbor any other recurrent mutations or chromosomal aberrations, high-grade NF2 mutant meningiomas generally harbor a more aberrant genome with several recurrent chromosomal gains and losses (including the loss of chr 4p, 6q, 7p, 9p, 10q, 11p, 14q, and 18q, and gain of chr 17q and 20q) in addition to functional NF2 inactivation/chromosome 22 loss. Frequently mutated genes in high-grade meningiomas include CDKN2A (mutated in 4% of atypical and 28% of anaplastic meningiomas), TERT promoter mutations (14 to 23% of WHO grade 3 meningiomas), ARID1A (5.4%), PTEN (4.3% and frequent loss of chromosome 10q harboring PTEN), KDM6A (3.5%), SUFU (2.7%), and TP53 (2.9%) (8–11).

Although considerably rarer compared to adult meningiomas, pediatric meningiomas do occur. Kirches et al. analyzed a cohort of 37 pediatric meningiomas and found that they were enriched for higher-grade tumors, and 30% of tumors were of WHO grade 1, 57% grade 2, and 14% of grade 3 (12). The most frequent genetic aberrations were loss of chromosome 22 (62%, harboring the NF2 gene) and loss of chromosomes 1, 14, and 18. The most common mutations (based on a targeted sequencing panel) were found in NF2, BRCA1, RGPD3, APC, TSC1, KDM6A, and SMARCE1; however, other mutations frequently found in adult meningiomas (TRAF7, KLF4, AKT1, SMO, and TERT) were either significantly underrepresented (TRAF7; 1 out of 34 tumors) or completely absent. Another study found an enrichment of YAP1 fusions in pediatric NF2 wild-type meningiomas (13). These YAP1 fusions constitute an alternative to NF2 loss and are oncogenic when expressed in mice, suggesting that they are the likely tumor-initiating events and oncogenic drivers in these tumors (14).

**Figure 1:**
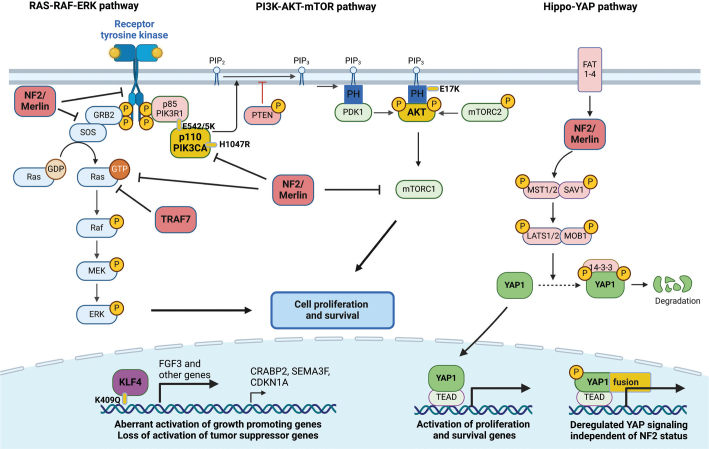
Overview schematic of the various primary mutations found in the different meningioma subtypes and the different pathways they affect. Created with BioRender.com.

In the following section, we will review the most common primary types of mutations found in meningioma (Figure 1).

### NF2/Merlin

#### Hippo pathway-dependent functions

The NF2 (Neurofibromatosis 2) gene encodes for the protein Merlin, is inactivated in around 40 to 60% of meningiomas, and is enriched in higher-grade tumors (5, 6). NF2 inactivation may occur via point mutations, truncations, or gene fusions, and up to 97% of patients who harbor sporadic NF2 mutations also exhibit an additional loss of chromosome 22q as the second hit. In addition, up to 50% of patients with germline NF2 mutations will develop one or multiple meningiomas during their lifetime, in addition to other tumors (most commonly Schwannomas). NF2/Merlin is a tumor suppressor and upstream regulator of the Hippo Signaling pathway that translates mechanical stimuli into transcriptional signals through a cascade of serine-threonine kinases that ultimately phosphorylate and inhibit the functions of the transcriptional coactivator YAP1 (Yes-associated protein 1, and its paralog TAZ) (15–19). Loss or functional inactivation of NF2 or other upstream Hippo Pathway mediators lead to deregulation and deinhibition of YAP1 and NF2 mutant meningiomas and/or cell lines express high levels of YAP activity and upregulate the expression of several canonical YAP1 targets (such as CTGF and CYR61) (14, 20, 21). Furthermore, the expression of a non-regulatable YAP1 variant (S127/397A-YAP1) induces very similar transcriptional changes compared to NF2 loss in human neural stem cells, and that the expression of the same construct in Nestin-positive cells in the meninges of *Cdkn2a* null mice induces the formation of meningioma-like tumors that resemble human NF2 mutant meningiomas by histomorphology and gene expression (14). Although to a rarer extent, additional Hippo Pathway inhibitors, such as FAT2 in spinal meningiomas (22), can also be mutated in meningioma, further highlighting the role of deregulated Hippo-YAP signaling in meningioma. The importance of deregulated YAP activity in the pathobiology of NF2 mutant meningioma is furthermore emphasized by the presence of recurrent YAP1 gene fusions (most frequently YAP1-MAML2) in around 10% of pediatric NF2 wild-type meningiomas (13, 14). The oncogenic functions of these YAP1 fusions fundamentally rely on their ability to exert deregulated YAP activity, and YAP1 fusion-positive human meningiomas resemble NF2 mutant meningiomas by both gene expression and DNA methylation-based classification (13–15, 23). Taken together, these findings mechanistically link oncogenic deregulated YAP activity to NF2 loss and meningioma pathobiology. It is currently unknown if low- and high-grade NF2 mutant meningiomas equally rely on YAP1 signaling for their growth and/or survival and would be susceptible to targeted therapy against YAP1. YAP1 is a transcriptional coactivator that does not bind DNA itself but relies on the interaction with other transcription factors (mostly TEADs1-4). Several pharmacological inhibitors of YAP signaling are currently being developed, most of them inhibiting the interaction of YAP1 with TEAD transcription factors, and their efficacy is being evaluated for other cancers, such as NF2 mutant mesothelioma (24). However, it remains unknown if these inhibitors are as effective against either low-grade meningiomas (that seem to largely rely on YAP signaling) or high-grade meningiomas (that harbor additional mutations and likely concurrently activate additional mitogenic pathways).

#### Hippo pathway-independent functions

In addition to the Hippo-YAP axis, NF2/Merlin also regulates the activity of other signaling pathways, including the RAS-RAF-MEK-ERK and PI3K-AKT-mTOR pathways, and other receptor tyrosine kinase (RTK) pathways, although this axis is less well understood to this date. Merlin has been shown to directly interact with RTKs and several RTK adapter proteins to modulate and inhibit their activity (25). For example, Merlin has been shown to interact with EGFR and block the trafficking of ligand-bound EGFR by sequestering it into a non-signaling membrane compartment, thereby inhibiting EGF-EGFR-mediated signaling (26). Merlin has also been shown to interact with and promote the degradation of PDGFR, ErbB2/ErbB3, and CD44 receptors (16, 27), as well as to inhibit Ras signaling, either by directly interacting with Ras or through inhibiting the functions of RasGEFs (such as Son of sevenless) or adapter proteins (Grb2) (28–30).

Merlin has also been shown to inhibit the PI3K-AKT-mTOR pathway in several ways. Merlin can directly inhibit the activity of PI3K by binding to PIKE-L, the GTPase that binds and activates PI3K, which, in turn, disrupts binding of PIKE-L to PI3K and inhibits PI3K (31). In addition, Merlin has also been shown to inhibit mTOR activation through PI3K-AKT-independent mechanisms (32). The frequency of AKT and PIK3CA mutations in NF2 wild-type meningiomas highlights a prominent role of this pathway in the pathobiology of meningiomas in general, and it is conceivable that the activation of these pathways may contribute to tumorigenesis also in NF2 mutant tumors.

### TRAF7

TRAF7 mutations are found in around 20–25% of meningiomas and most frequently occur in the C-terminal WD40 repeat domain. TRAF7 mutations are virtually mutually exclusive with NF2 mutations but frequently cooccur with either KLF4, AKT, or PIK3CA mutations. TRAF7 is an E3 ubiquitin ligase that was initially classified as a member of the TNF receptor-associated factor (TRAF) family. Although it shares several features with other TRAF family members, such as an N-terminal RING finger domain, it lacks the classical C-terminal TRAF domain and instead contains a WD40 repeat domain responsible for protein–protein interactions (33). Like other TRAF family members, TRAF7 was originally identified as a component of the NF-κB pathway. In addition, TRAF7 has been shown to promote the ubiquitination of several proteins, including NEMO, the p65 subunit of NF-κB transcription factor, the anti-apoptotic protein c-FLIP (CFLAR), and the tumor suppressor protein p53 (TP53) (33). More recent findings have shown that TRAF7 interacts and inhibits the function of several Ras-related GTPases in meningeal cells via its WD40 domain, including HRas, KRas, CDC42, RAC1/2/3, and RHOA (34). The most recurrent WD40 domain TRAF7 mutants found in meningioma displayed a markedly reduced ability to bind either CDC42 or HRas, whereas mutations in the RING catalytic domain of TRAF7 (such as TRAF7-R153S) had no effect on CDC42 or HRas binding. Loss of TRAF7 in meningeal cells promoted the activity of CDC42 and the p21-activated kinase PAK1 and enhanced RAS activity and the upregulation of the MAPK cascade in meningioma cells, suggesting that these pathways might be important in TRAF7-mutated meningiomas (34).

### KLF4

KLF4 (Krüppel-like factor 4) is a transcription factor involved in regulating cellular differentiation and proliferation and belonged to one of four transcription factors – together with SOX2, OCT4, and c-Myc – used for the reprogramming of adult somatic cells into induced pluripotent stem cells (iPSCs) (35, 36). KLF4 belongs to the Specificity protein (Sp) and KLF (Sp/KLF) transcription factor superfamily that is characterized by the presence of three C2H2 zinc fingers within the DNA-binding domain. KLF4-K409Q mutations are found in a subset of NF2 wild-type meningiomas and almost always cooccur with mutations in TRAF7. The K409Q mutation lies in the first zinc finger of the KLF4 DNA-binding domain, and the lysine residue in this position is conserved between all members of the Sp/KLF superfamily (37). The exact impact of this mutation remains unknown, and both gain- and loss-of-function effects have been reported. Tsytsykova et al. reported that the K409Q mutation alters the DNA recognition preference of KLF4, resulting in a shift in downstream transcriptional activity. Among other targets, this leads to the transcriptional activation of fibroblast growth factor 3 (FGF3) expression in KLF4-K409Q-expressing cells, not present in wild-type KLF4-expressing cells, and KLF4-K409Q-mutated meningiomas expressed higher levels of FGF3 compared to KLF4-wild-type tumors (37). Furthermore, KLF4-K409Q-expressing meningioma cells show high levels of PI3K-AKT-mTOR pathway activation and respond to mTOR inhibitors (38). Lastly, the activation of the Ras pathway caused by loss or mutation of TRAF7 results in KLF4-mediated growth inhibition in KLF4 wild-type cells, in part by the KLF4-mediated transcriptional activation of several growth inhibiting genes, such as CRABP2, SEMA3F, and CDKN1A (34). The KLF4-K409Q mutant was unable to recapitulate these growth inhibitory functions of wild-type KLF4, supplying a partial rational for the frequent cooccurrence of TRAF7 and KLF4 mutations.

### Mutations in PI3K-AKT-mTOR pathway members

NF2 wild-type meningiomas are also enriched for ‘driver mutations’ in members of the PI3K-AKT-mTOR pathway, most commonly PIK3CA, AKT1, and to a lesser extent PIK3R1 (7, 39–41). These mutations lead to aberrant PI3K pathway activation and often cooccur with TRAF7 mutations but are mutually exclusive from NF2 mutations and from each other. In addition, functional inactivation of PTEN (a negative regulator of the PI3K pathway; frequently achieved by loss of chromosome 10) is also enriched in higher-grade meningiomas (most frequently NF2 mutant).

The PI3K pathway is one of the most important intracellular pathways, regulating cell growth, motility, and survival and transmits signals from several receptor types, including RTKs, G protein-coupled receptors, and small Ras-related GTPases (42, 43). Upon receptor activation, the plasma membrane-associated lipid kinase PI3K is recruited to the receptor and catalyzes the phosphorylation of phosphatidylinositol-4,5-bisphosphate (PIP2) to produce phosphatidylinositol-3,4,5-trisphosphate (PIP3), a second messenger inositol that binds to the pleckstrin-homology (PH) domain of the serine/threonine kinase AKT, thereby localizing AKT and its upstream activating kinase PDK-1 to the cell membrane (44). Phosphorylation of AKT at Thr308 by PDK-1 and subsequent phosphorylation at Ser473 by mTORC2 leads to its full activation. Activated AKT then phosphorylates many substrates involved in cell proliferation, metabolism, survival, and motility.

PI3K consists of three subunits: the p110 catalytic subunit (p110α is encoded by PIK3CA), the p85 regulatory subunit (p85α is encoded by PIK3R1), and the p55 regulatory subunit (42, 43). Several different PIK3CA activating mutations have been identified in meningioma and other tumors, with H1047R and E542K/E545K as the two most common mutation ‘hotspots’ (39, 43). The H1047R mutation enhances the interaction of the kinase domain with the plasma membrane and eliminates the requirement for association with Ras, whereas the E542K and E545K mutations disrupt the interaction with the p55 and p85 regulatory subunits. Mutations in PIK3R1 frequently disrupt binding of the p85α regulatory subunit to p110.

Mutations in the PI3K effector AKT1 (AKT1-E17K) are located in the PH domain of AKT1 and increase the affinity of AKT1 to PIP2 by 100-fold and that of PIP3 by 7-fold, resulting in enhanced AKT activity and activation of PI3K-AKT signaling (44).

The importance of the PI3K-AKT-mTOR pathway in the pathobiology of meningiomas is furthermore emphasized by the fact that chr 10q, harboring the PTEN gene, an inhibitor of this pathway, is frequently lost in high-grade NF2 mutant meningiomas. In addition to its functions as an inhibitor of the PI3K pathway, PTEN has also been shown to elicit tumor suppressor functions in the nucleus in various ways, for example, by directly binding to chromatin in promoter and putative enhancer regions and negatively regulating the expression of genes involved in transcription (45). However, the different roles of PTEN function in the pathogenesis of meningioma have so far not been studied in detail.

### Novel classifications based on epigenetic markers and next generation sequencing efforts

The WHO grade system does not always accurately identify high-grade meningiomas, and better classification systems are needed to infer patient prognosis. Currently available standard-of-care therapeutic options are limited to surgery and radiation therapy; therefore, the underlying biology of meningioma subtypes needs to be better understood to develop effective medical therapies. To address these gaps in the field, multiple groups have introduced next generation sequencing-based analysis focusing on DNA copy number aberrations, DNA point mutations, DNA methylation, and RNA quantification (6, 46–49).

Genome-wide DNA methylation patterns were one of the earliest molecular signatures used to characterize and classify meningiomas as an alternative to histopathological based grading (6). Meningiomas were initially classified into two major categories of benign and malignant tumors and were later further subdivided into six subgroups (four benign and two malignant) using unsupervised clustering of methylation patterns (6, 50). Mutations identified using whole exome or whole genome sequencing added another layer of information defining a more robust classification of meningioma. NF2 mutations were significantly enriched in all subtypes except for the MC ben-2 subtype, which, in turn, was the only methylation subtype enriched for mutations in TRAF7, AKT1, SMO, and KLF4. Such integrative molecular classification using methylation patterns, copy number alterations, and RNA expression put forth by Nassiri et al. identified four major meningioma molecular groups (46). While tumors in the MG1, MG3, and MG4 subgroups were enriched for NF2 mutations, MG2 tumors were relatively benign and harbor wild-type NF2 but were enriched for TRAF7, KLF4, and AKT1 mutations. These molecular groups are further distinguished by underlying biology that may inform on new therapeutics. MG1 was enriched for immunogenic signatures, while MG3 and MG4 were enriched for hypermetabolic and proliferation related gene expression, respectively. Chromosome level disruptions were also added onto subtypes to strengthen the classification of tumors. MG1 tumors were found to be relatively diploid except for loss of chromosome 22q. MG2 subtype further divided into two groups that were mutually exclusive for mutations and chromosome alterations. MG3 and MG4, being the most aggressive, were high aneuploidy tumors (losses of multiple chromosomes, e.g., 22q, 1p, 6q, 14, 18, and 10). A similar methylation and copy number-based classification was developed by David Raleigh’s group at UCSF (47). They identify three major meningioma classes, namely, Merlin-intact, Immune-enriched, and Hypermitotic, which comply with MG2, MG1, and MG3/4 of the previously described study, respectively.

One benefit of RNA-Seq over the other molecular data types is that it allows for direct insight into the activated pathways in the analyzed tumors, albeit the drawback that tumor bulk sequencing includes transcriptional signals from microenvironmental and other non-tumor cells in the tissue. This approach provides insight into underlying biological signature of different meningioma subclasses. RNA-Seq data from large numbers of human meningiomas when displayed in a UMAP shows significant correlation between expression patterns, and clinical and genomic data, such as tumor grade, time to recurrence, functional NF2 status. Further such RNA seq based UMAPs correlate other classification strategies such as DNA methylation-based classifications with regions of the reference map. In addition, because general regions across the map correlate with specific biological signatures and different patient outcomes, map location may be useful in predicting tumor biology and patient prognosis (51).

These recent applications of next-generation sequencing-based approaches identify new biomarkers and oncogenic drivers and may serve to craft therapeutic targets in the scope of precision medicine.

### Preclinical mouse models of meningioma

Preclinical mouse models are invaluable tools for the in vivo testing of novel agents, since the rarity of certain tumor types, for example, high-grade meningiomas (and specific subtypes thereof), makes it difficult to recruit sufficient numbers of patients for randomized, large clinical trials. Only recently have we begun to characterize and understand the genetic and molecular drivers of different meningioma subtypes, and further stratification would be necessary for targeted therapies. Thus, preclinical models that, on the one hand, accurately replicate patient biology but, on the other hand, are genetically defined are critical for developing novel targeted therapeutics.

In general, mouse models can be distinguished into transplantation models, xenograft or allograft injections of human or mouse tumor cells, and GEMMs, in which a tumor forms de novo in mice, for example, caused by the exogenous expression of an oncogenic driver (52). Several cell line and patient-derived xenograft (PDX) meningioma models exist (reviewed in detail in (53) and (54)). However, until recently, reliable meningioma GEMMs were lacking. Each model type has specific advantages and disadvantages. PDX meningioma models have the advantage that these are actual human tumor cells that harbor and are driven by actual mutations found in meningioma. On the other hand, PDX models can only be grown in vivo in immune-compromised mice, and these tumors oftentimes activate graft-versus-host programs in myeloid cells, limiting their value for studies investigating immune cells or testing immunotherapy approaches. By contrast, GEMMs allow for the specific development of genetically defined tumor models; however, they rely on the presence of strong oncogenic drivers. The genetically defined nature of these models allows for a defined test population; however, these models might also fail to recapitulate the genetic variety and the interplay between different mutations and several oncogenic drivers present in actual human tumor cells if these are not present in the model. Furthermore, gains and losses of entire chromosomes (found in high-grade meningiomas) are currently impossible to model in GEMMs largely because there is considerable shuffling of the DNA order both within and between chromosomes when comparing human and mouse genomes. Therefore, GEMMs are useful tools to model and assess the oncogenic potential of specific and selected tumor oncogenes and suppressors, as well as to test targeted therapies against these mutations, but might fail to recapitulate every aspect of a specific cancer and the influence of the entire aberrant genome as a whole.

The lack of known strong oncogenic drivers in meningioma has hindered the development of reliable meningioma GEMMs until recently. The Kalamarides lab has previously developed several meningioma GEMMs relying on the Adenovirus Cre (AdCre)-mediated deletion of the Nf2 gene in PGDS (Prostaglandin D synthase)-expressing arachnoid cap cells in mice either in a wild-type or Cdkn2a null background (55, 56). Nf2 deletion in a wild-type background resulted in the formation of meningioma-like tumors in 19% (subdural injection, median time to tumor was 14 months) and 29% of mice (transorbital injection, median time to tumor was 11 months). Additional Cdkn2a deletion (lost in a subset of high-grade meningiomas) leads to an increased frequency of meningioma-like tumors (72%) and a shorter latency (3.5 months). However, local extravasation of AdCre during injection leads to the early development of subcutaneous sarcomas and aggressive liver tumors in a large percentage of mice.

Finally, we have recently developed two models of adult and pediatric meningiomas using the RCAS/tv-a system for postnatal somatic-cell gene transfer (14, 57). RCAS (Replication-Competent ASLV long terminal repeat [LTR]) is an ASLV-based retrovirus that can only infect mammalian cells that have been engineered to express the viral tv-a receptor. The RCAS-mediated expression of either YAP1-MAML2 (found in a subset of pediatric NF2 wild-type meningiomas) or S127/397A-YAP1 (a constitutively active form of YAP1 that mimics functional NF2 loss) in Nestin-positive cells in the meninges of Cdkn2a null new-born mouse pups resulted in the formation of high-grade meningioma-like tumors in 68 and 97% of mice, respectively, with a latency of 80–150 days. These tumors frequently grew as extra-axial, extracranial, and intraventricular meningiomas and responded to YAP-directed therapy ex vivo.

The advantage of the RCAS/tv-a system is its versatility. Since the oncogenic driver is introduced by the RCAS virus, and not via germline genome engineering, this allows for the rapid exchange and/or modification of the oncogene and the expression of several oncogenes (expressed via the same or via multiple RCAS vectors) or the in vivo knockdown or knockout of tumor suppressor genes (57). This will allow for the relatively rapid evaluation of different oncogenic drivers and tumor suppressor losses found in high-grade meningiomas and their influence on tumor latency, biology, and response to therapy.

## Conclusion and outlook

Despite their relative frequency, meningiomas have been an understudied disease, largely due to the fact that the majority of these tumors are frequently benign and curable by surgery alone due to their noninvasive nature and the scarcity of reliable experimental in vivo systems for preclinical testing. However, tumors in difficult locations (such as skull base meningiomas) or high-grade meningiomas that present with brain invasion and frequently recur despite several rounds of surgery and/or radiation therapy highlight the need for a better understanding of the biology of these tumors and their oncogenic drivers. This is especially true for high-grade meningiomas that frequently harbor a multitude of additional mutations that lead to a multitude of concurrently activated pathways similar to other high-grade tumors, such as gliomas, rendering the treatment of single specific pathways infeasible.

Recent next-generation sequencing efforts have greatly added to our understanding of the meningioma landscape and the underlying driver mutations and have yielded new classification tools that can lead to better survival predictions compared to histopathological grading alone. Although our growing knowledge of the genetic landscape of meningiomas has so far not translated into effective novel therapeutic approaches, the identification of novel targets and the stratification of patients based on the genetic markup of their tumors will ultimately help improve patient outcomes, and several new clinical trials (such as FAK (Focal adhesion kinase) inhibitors for SMO- or NF2-mutant meningiomas) are currently underway. Preclinical mouse models of meningioma will be invaluable tools to assess the impact of specific mutations on tumor biology and aggressiveness and to test the efficacy of specific novel inhibitors before advancing into clinical trials with patients.

## References

[1] Ostrom QT, Price M, Neff C, Cioffi G, Waite KA, Kruchko C, et al. CBTRUS statistical report: primary brain and other central nervous system tumors diagnosed in the United States in 2015–2019. Neuro Oncol. 2022;24:v1–95. doi: 10.1093/neuonc/noac202 36196752 PMC9533228

[2] Wang JZ, Nassiri F, Saladino A, Zadeh G. Surgical therapy of non-skull base meningiomas. Adv Exp Med Biol. 2023;1416:79–94. doi: 10.1007/978-3-031-29750-2_7 37432621

[3] Westphal M, Saladino A, Tatagiba M. Skull base meningiomas. Adv Exp Med Biol. 2023;1416:47–68. doi: 10.1007/978-3-031-29750-2_5 37432619

[4] Louis DN, Perry A, Wesseling P, Brat DJ, Cree IA, Figarella-Branger D, et al. The 2021 WHO classification of tumors of the central nervous system: a summary. Neuro Oncol. 2021;23:1231–51. doi: 10.1093/neuonc/noab106 34185076 PMC8328013

[5] Wang JZ, Nassiri F, Mawrin C, Zadeh G. Genomic landscape of meningiomas. Adv Exp Med Biol. 2023;1416:137–58. doi: 10.1007/978-3-031-29750-2_11 37432625

[6] Sahm F, Schrimpf D, Stichel D, Jones DTW, Hielscher T, Schefzyk S, et al. DNA methylation-based classification and grading system for meningioma: a multicentre, retrospective analysis. Lancet Oncol. 2017;18:682–94. doi: 10.1016/S1470-2045(17)30155-9 28314689

[7] Clark VE, Erson-Omay EZ, Serin A, Yin J, Cotney J, Ozduman K, et al. Genomic analysis of non-NF2 meningiomas reveals mutations in TRAF7, KLF4, AKT1, and SMO. Science. 2013;339:1077–80. doi: 10.1126/science.1233009 23348505 PMC4808587

[8] Sievers P, Hielscher T, Schrimpf D, Stichel D, Reuss DE, Berghoff AS, et al. CDKN2A/B homozygous deletion is associated with early recurrence in meningiomas. Acta Neuropathol. 2020;140:409–13. doi: 10.1007/s00401-020-02188-w 32642869 PMC7423850

[9] Williams EA, Santagata S, Wakimoto H, Shankar GM, Barker FG, 2nd, Sharaf R, et al. Distinct genomic subclasses of high-grade/progressive meningiomas: NF2-associated, NF2-exclusive, and NF2-agnostic. Acta Neuropathol Commun. 2020;8:171. doi: 10.1186/s40478-020-01040-2 33087175 PMC7580027

[10] Spiegl-Kreinecker S, Lotsch D, Neumayer K, Kastler L, Gojo J, Pirker C, et al. TERT promoter mutations are associated with poor prognosis and cell immortalization in meningioma. Neuro Oncol. 2018;20:1584–93. doi: 10.1093/neuonc/noy104 30010853 PMC6231195

[11] Goutagny S, Nault JC, Mallet M, Henin D, Rossi JZ, Kalamarides M. High incidence of activating TERT promoter mutations in meningiomas undergoing malignant progression. Brain Pathol. 2014;24:184–9. doi: 10.1111/bpa.12110 24261697 PMC8029399

[12] Kirches E, Sahm F, Korshunov A, Bluecher C, Waldt N, Kropf S, et al. Molecular profiling of pediatric meningiomas shows tumor characteristics distinct from adult meningiomas. Acta Neuropathol. 2021;142:873–86. doi: 10.1007/s00401-021-02351-x 34495383 PMC8500891

[13] Sievers P, Chiang J, Schrimpf D, Stichel D, Paramasivam N, Sill M, et al. YAP1-fusions in pediatric NF2-wildtype meningioma. Acta Neuropathol. 2020;139:215–8. doi: 10.1007/s00401-019-02095-9 31734728

[14] Szulzewsky F, Arora S, Arakaki AKS, Sievers P, Almiron Bonnin DA, Paddison PJ, et al. Both YAP1-MAML2 and constitutively active YAP1 drive the formation of tumors that resemble NF2 mutant meningiomas in mice. Genes Dev. 2022;36:857–70. doi: 10.1101/gad.349876.122 36008139 PMC9480855

[15] Szulzewsky F, Holland EC, Vasioukhin V. YAP1 and its fusion proteins in cancer initiation, progression and therapeutic resistance. Dev Biol. 2021;475:205–21. doi: 10.1016/j.ydbio.2020.12.018 33428889 PMC8107117

[16] Petrilli AM, Fernandez-Valle C. Role of Merlin/NF2 inactivation in tumor biology. Oncogene. 2016;35:537–48. doi: 10.1038/onc.2015.125 25893302 PMC4615258

[17] Hamaratoglu F, Willecke M, Kango-Singh M, Nolo R, Hyun E, Tao C, et al. The tumour-suppressor genes NF2/Merlin and expanded act through Hippo signalling to regulate cell proliferation and apoptosis. Nat Cell Biol. 2006;8:27–36. doi: 10.1038/ncb1339 16341207

[18] Yin F, Yu J, Zheng Y, Chen Q, Zhang N, Pan D. Spatial organization of Hippo signaling at the plasma membrane mediated by the tumor suppressor Merlin/NF2. Cell. 2013;154:1342–55. doi: 10.1016/j.cell.2013.08.025 24012335 PMC3835333

[19] Zhang N, Bai H, David KK, Dong J, Zheng Y, Cai J, et al. The Merlin/NF2 tumor suppressor functions through the YAP oncoprotein to regulate tissue homeostasis in mammals. Dev Cell. 2010;19:27–38. doi: 10.1016/j.devcel.2010.06.015 20643348 PMC2925178

[20] Baia GS, Caballero OL, Orr BA, Lal A, Ho JS, Cowdrey C, et al. Yes-associated protein 1 is activated and functions as an oncogene in meningiomas. Mol Cancer Res. 2012;10:904–13. doi: 10.1158/1541-7786.MCR-12-0116 22618028 PMC4703097

[21] Striedinger K, VandenBerg SR, Baia GS, McDermott MW, Gutmann DH, Lal A. The neurofibromatosis 2 tumor suppressor gene product, merlin, regulates human meningioma cell growth by signaling through YAP. Neoplasia. 2008;10:1204–12. doi: 10.1593/neo.08642 18953429 PMC2570596

[22] Tate G, Kishimoto K, Mitsuya T. A novel mutation of the FAT2 gene in spinal meningioma. Oncol Lett. 2016;12:3393–6. doi: 10.3892/ol.2016.5063 27900010 PMC5103956

[23] Szulzewsky F, Arora S, Hoellerbauer P, King C, Nathan E, Chan M, et al. Comparison of tumor-associated YAP1 fusions identifies a recurrent set of functions critical for oncogenesis. Genes Dev. 2020;34:1051–64. doi: 10.1101/gad.338681.120 32675324 PMC7397849

[24] Study to evaluate VT3989 in patients with metastatic solid tumors enriched for tumors with NF2 gene mutations: ClinicalTrials.gov; 2021. Available from: https://clinicaltrials.gov/study/NCT04665206 [cited February 2024].

[25] Stamenkovic I, Yu Q. Merlin, a ‘magic’ linker between extracellular cues and intracellular signaling pathways that regulate cell motility, proliferation, and survival. Curr Protein Pept Sci. 2010;11:471–84. doi: 10.2174/138920310791824011 20491622 PMC2946555

[26] Chiasson-MacKenzie C, Morris ZS, Baca Q, Morris B, Coker JK, Mirchev R, et al. NF2/Merlin mediates contact-dependent inhibition of EGFR mobility and internalization via cortical actomyosin. J Cell Biol. 2015;211:391–405. doi: 10.1083/jcb.201503081 26483553 PMC4621825

[27] Fraenzer JT, Pan H, Minimo L, Jr., Smith GM, Knauer D, Hung G. Overexpression of the NF2 gene inhibits schwannoma cell proliferation through promoting PDGFR degradation. Int J Oncol. 2003;23:1493–500. doi: 10.3892/ijo.23.6.1493 14612918

[28] Cui Y, Groth S, Troutman S, Carlstedt A, Sperka T, Riecken LB, et al. The NF2 tumor suppressor merlin interacts with Ras and RasGAP, which may modulate Ras signaling. Oncogene. 2019;38:6370–81. doi: 10.1038/s41388-019-0883-6 31312020 PMC6756068

[29] Morrison H, Sperka T, Manent J, Giovannini M, Ponta H, Herrlich P. Merlin/neurofibromatosis type 2 suppresses growth by inhibiting the activation of Ras and Rac. Cancer Res. 2007;67:520–7. doi: 10.1158/0008-5472.CAN-06-1608 17234759

[30] Lim JY, Kim H, Jeun SS, Kang SG, Lee KJ. Merlin inhibits growth hormone-regulated Raf-ERKs pathways by binding to Grb2 protein. Biochem Biophys Res Commun. 2006;340:1151–7. doi: 10.1016/j.bbrc.2005.12.122 16405865

[31] Rong R, Tang X, Gutmann DH, Ye K. Neurofibromatosis 2 (NF2) tumor suppressor merlin inhibits phosphatidylinositol 3-kinase through binding to PIKE-L. Proc Natl Acad Sci U S A. 2004;101:18200–5. doi: 10.1073/pnas.0405971102 15598747 PMC535703

[32] James MF, Han S, Polizzano C, Plotkin SR, Manning BD, Stemmer-Rachamimov AO, et al. NF2/merlin is a novel negative regulator of mTOR complex 1, and activation of mTORC1 is associated with meningioma and schwannoma growth. Mol Cell Biol. 2009;29:4250–61. doi: 10.1128/MCB.01581-08 19451225 PMC2715803

[33] Zotti T, Scudiero I, Vito P, Stilo R. The emerging role of TRAF7 in tumor development. J Cell Physiol. 2017;232:1233–8. doi: 10.1002/jcp.25676 27808423 PMC5347962

[34] Najm P, Zhao P, Steklov M, Sewduth RN, Baietti MF, Pandolfi S, et al. Loss-of-function mutations in TRAF7 and KLF4 cooperatively activate RAS-Like GTPase signaling and promote meningioma development. Cancer Res. 2021;81:4218–29. doi: 10.1158/0008-5472.CAN-20-3669 34215617

[35] Takahashi K, Tanabe K, Ohnuki M, Narita M, Ichisaka T, Tomoda K, et al. Induction of pluripotent stem cells from adult human fibroblasts by defined factors. Cell. 2007;131:861–72. doi: 10.1016/j.cell.2007.11.019 18035408

[36] Takahashi K, Yamanaka S. Induction of pluripotent stem cells from mouse embryonic and adult fibroblast cultures by defined factors. Cell. 2006;126:663–76. doi: 10.1016/j.cell.2006.07.024 16904174

[37] Tsytsykova AV, Wiley G, Li C, Pelikan RC, Garman L, Acquah FA, et al. Mutated KLF4(K409Q) in meningioma binds STRs and activates FGF3 gene expression. iScience. 2022;25:104839. doi: 10.1016/j.isci.2022.104839 35996584 PMC9391581

[38] von Spreckelsen N, Waldt N, Poetschke R, Kesseler C, Dohmen H, Jiao HK, et al. KLF4(K409Q)-mutated meningiomas show enhanced hypoxia signaling and respond to mTORC1 inhibitor treatment. Acta Neuropathol Commun. 2020;8:41. doi: 10.1186/s40478-020-00912-x 32245394 PMC7118946

[39] Abedalthagafi M, Bi WL, Aizer AA, Merrill PH, Brewster R, Agarwalla PK, et al. Oncogenic PI3K mutations are as common as AKT1 and SMO mutations in meningioma. Neuro Oncol. 2016;18:649–55. doi: 10.1093/neuonc/nov316 26826201 PMC4827048

[40] Clark VE, Harmanci AS, Bai H, Youngblood MW, Lee TI, Baranoski JF, et al. Recurrent somatic mutations in POLR2A define a distinct subset of meningiomas. Nat Genet. 2016;48:1253–9. doi: 10.1038/ng.3651 27548314 PMC5114141

[41] Youngblood MW, Duran D, Montejo JD, Li C, Omay SB, Ozduman K, et al. Correlations between genomic subgroup and clinical features in a cohort of more than 3000 meningiomas. J Neurosurg. 2019;133(5): 1345–54. doi: 10.3171/2019.8.JNS191266 31653806

[42] Yang J, Nie J, Ma X, Wei Y, Peng Y, Wei X. Targeting PI3K in cancer: mechanisms and advances in clinical trials. Mol Cancer. 2019;18:26. doi: 10.1186/s12943-019-0954-x 30782187 PMC6379961

[43] Fruman DA, Chiu H, Hopkins BD, Bagrodia S, Cantley LC, Abraham RT. The PI3K pathway in human disease. Cell. 2017;170:605–35. doi: 10.1016/j.cell.2017.07.029 28802037 PMC5726441

[44] Chen Y, Huang L, Dong Y, Tao C, Zhang R, Shao H, et al. Effect of AKT1 (p. E17K) hotspot mutation on malignant tumorigenesis and prognosis. Front Cell Dev Biol. 2020;8:573599. doi: 10.3389/fcell.2020.573599 33123537 PMC7573235

[45] Steinbach N, Hasson D, Mathur D, Stratikopoulos EE, Sachidanandam R, Bernstein E, et al. PTEN interacts with the transcription machinery on chromatin and regulates RNA polymerase II-mediated transcription. Nucleic Acids Res. 2019;47:5573–86. doi: 10.1093/nar/gkz272 31169889 PMC6582409

[46] Nassiri F, Liu J, Patil V, Mamatjan Y, Wang JZ, Hugh-White R, et al. A clinically applicable integrative molecular classification of meningiomas. Nature. 2021;597:119–25. doi: 10.1038/s41586-021-03850-3 34433969 PMC11604310

[47] Choudhury A, Magill ST, Eaton CD, Prager BC, Chen WC, Cady MA, et al. Meningioma DNA methylation groups identify biological drivers and therapeutic vulnerabilities. Nat Genet. 2022;54:649–59. doi: 10.1038/s41588-022-01061-8 35534562 PMC9374001

[48] Choudhury A, Chen WC, Lucas CG, Bayley JC, Harmanci AS, Maas SLN, et al. Hypermitotic meningiomas harbor DNA methylation subgroups with distinct biological and clinical features. Neuro Oncol. 2023;25:520–30. doi: 10.1093/neuonc/noac224 36227281 PMC10013643

[49] Prager BC, Vasudevan HN, Dixit D, Bernatchez JA, Wu Q, Wallace LC, et al. The meningioma enhancer landscape delineates novel subgroups and drives druggable dependencies. Cancer Discov. 2020;10:1722–41. doi: 10.1158/2159-8290.CD-20-0160 32703768 PMC8194360

[50] Olar A, Wani KM, Wilson CD, Zadeh G, DeMonte F, Jones DT, et al. Global epigenetic profiling identifies methylation subgroups associated with recurrence-free survival in meningioma. Acta Neuropathol. 2017;133:431–44. doi: 10.1007/s00401-017-1678-x 28130639 PMC5600514

[51] Thirimanne HN, Bonnin DA, Nuechterlein N, Arora S, Jensen M, Parada CA, et al. Meningioma transcriptomic landscape demonstrates novel subtypes with regional associated biology and patient outcome. bioRxiv. 2024:2024.02.23.581766. doi: 10.1101/2024.02.23.581766 PMC11228955 38788713

[52] Arakaki AKS, Szulzewsky F, Gilbert MR, Gujral TS, Holland EC. Utilizing preclinical models to develop targeted therapies for rare central nervous system cancers. Neuro Oncol. 2021;23:S4–15. doi: 10.1093/neuonc/noab183 34725698 PMC8561121

[53] Khan M, Hanna C, Findlay M, Lucke-Wold B, Karsy M, Jensen RL. Modeling meningiomas: optimizing treatment approach. Neurosurg Clin N Am. 2023;34:479–92. doi: 10.1016/j.nec.2023.02.014 37210136

[54] Jungwirth G, Hanemann CO, Dunn IF, Herold-Mende C. Preclinical models of meningioma. Adv Exp Med Biol. 2023;1416:199–211. doi: 10.1007/978-3-031-29750-2_15 37432629

[55] Peyre M, Stemmer-Rachamimov A, Clermont-Taranchon E, Quentin S, El-Taraya N, Walczak C, et al. Meningioma progression in mice triggered by Nf2 and Cdkn2ab inactivation. Oncogene. 2013;32:4264–72. doi: 10.1038/onc.2012.436 23045274

[56] Kalamarides M, Niwa-Kawakita M, Leblois H, Abramowski V, Perricaudet M, Janin A, et al. Nf2 gene inactivation in arachnoidal cells is rate-limiting for meningioma development in the mouse. Genes Dev. 2002;16:1060–5. doi: 10.1101/gad.226302 12000789 PMC186259

[57] Kanvinde PP, Malla AP, Connolly NP, Szulzewsky F, Anastasiadis P, Ames HM, et al. Leveraging the replication-competent avian-like sarcoma virus/tumor virus receptor-A system for modeling human gliomas. Glia. 2021;69:2059–76. doi: 10.1002/glia.23984 33638562 PMC8591561

